# Waterproof Cellulose-Based Substrates for In-Drop Plasmonic Colorimetric Sensing of Volatiles: Application to Acid-Labile Sulfide Determination in Waters

**DOI:** 10.1021/acssensors.1c02585

**Published:** 2022-03-14

**Authors:** Nerea Villarino, Francisco Pena-Pereira, Isela Lavilla, Carlos Bendicho

**Affiliations:** Centro de Investigación Mariña, Universidade de Vigo, Departamento de Química Analítica e alimentaria, Grupo QA2, Edificio CC Experimentais, Campus de Vigo, As Lagoas, Marcosende, 36310 Vigo, Spain

**Keywords:** waterproof paper, microextraction, paper-based analytical devices, plasmonic sensing, smartphone-based detection, sulfide, waters

## Abstract

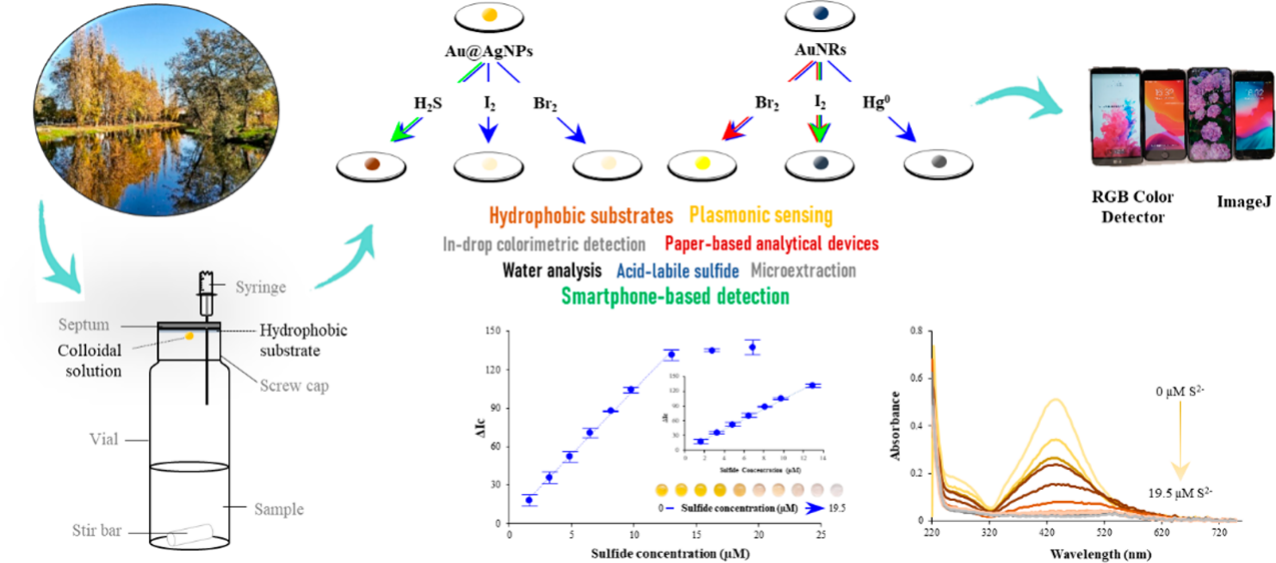

The present work reports on the assessment of widely available waterproof cellulose-based substrates for the development of sensitive in-drop plasmonic sensing approaches. The applicability of three inexpensive substrates, namely, Whatman 1PS, polyethylene-coated filter paper, and tracing paper, as holders for microvolumes of colloidal solutions was evaluated. Waterproof cellulose-based substrates demonstrated to be highly convenient platforms for analytical purposes, as they enabled *in situ* generation of volatiles and syringeless drop exposure unlike conventional single-drop microextraction approaches and can behave as sample compartments for smartphone-based colorimetric sensing in an integrated way. Remarkably, large drop volumes (≥20 μL) of colloidal solutions can be employed for enrichment processes when using Whatman 1PS as holder. In addition, the stability and potential applicability of spherical, rod-shaped, and core–shell metallic NPs onto waterproof cellulose-based substrates was evaluated. In particular, Au@AgNPs showed potential for the colorimetric detection of *in situ* generated H_2_S, I_2_, and Br_2_, whereas AuNRs hold promise for I_2_, Br_2_, and Hg^0^ colorimetric sensing. As a proof of concept, a smartphone-based colorimetric assay for determination of acid-labile sulfide in environmental water samples was developed with the proposed approach taking advantage of the ability of Au@AgNPs for H_2_S sensing. The assay showed a limit of detection of 0.46 μM and a repeatability of 4.4% (*N* = 8), yielding satisfactory recoveries (91–107%) when applied to the analysis of environmental waters.

The development of analytical strategies enabling a straightforward, selective, and sensitive determination of target compounds by means of plasmonic nanoparticles (NPs) is a challenging task that can be tackled by the combination of miniaturized analytical approaches.^1−3^ Of particular interest is the implementation of NPs in headspace microseparation approaches, since they potentially provide excellent enrichment factors with reduced matrix effects, while avoiding compatibility issues of NPs with complex samples. In this sense, a number of approaches have been reported in the literature to perform in-drop extraction of volatiles with simultaneous or sequential colorimetric detection. Downsized extraction approaches combined with miniaturized UV–vis spectrophotometers^2,4−6^ and probes^7,8^ have demonstrated their convenience for determination of target analytes at trace levels. More recently, nonconventional detection devices such as digital cameras and smartphones have received much interest for on-site analysis, mainly in combination with paper-based analytical devices (PADs).^9−13^ These systems proved suitable when combined with both drops and cellulose substrates as platforms for enrichment of volatiles with subsequent colorimetric detection.^14−17^ However, the use of PADs modified with plasmonic NPs for analyte sensing suffers from certain limitations, including potential aggregation and nonuniform distribution issues, as well as a reduced retention efficiency of NPs on cellulose substrates.^18^ While certain NPs are conveniently retained in PADs by drop casting,^19^ the retention of NPs at the surface of cellulose substrates is commonly limited, leading to faintly colored detection areas in PADs^20^ that can affect their sensitivity when applied for colorimetric sensing. This drawback is usually minimized by immersion of cellulose substrates in colloidal solutions for extended exposure times or via vacuum filtration,^21^ in spite of the relatively large amounts of colloidal solutions commonly required. NP-modified PADs can also be obtained by the drop-casting/heating approach^22^ or by *in situ* formation of NPs in the detection areas of PADs.^22^ The *in situ* synthesis of certain NPs with controlled sizes and morphologies in cellulose substrates is however a challenging task. Alternatively, colloidal solutions have been employed as extractant phases in single-drop microextraction (SDME), enabling the enrichment of volatiles with a reduced consumption of colloidal solution, typically in the range of 1–5 μL. However, the deposition of enriched microdrops on hydrophilic cellulose substrates for acquisition of the analytical signal results in a loss of sensitivity due to the aforementioned reduced retention of NPs on these materials. A remarkable and straightforward alternative involves the use of Eppendorf tubes for exposure of a drop of colloidal solution hanging from the cap with subsequent digitization for colorimetric analysis.^23^ This approach, however, hinders the *in situ* generation of volatiles from nonvolatile analytes without losses of gaseous derivatives, and convection is not produced during extraction, thus potentially leading to extended extraction times and/or limited sensitivity. Solving the aforementioned limitations, while integrating steps, would be therefore highly desirable.

Cellulose substrates display excellent properties such as capillary-driven fluid transport, flexibility, and low cost that make them ideal for a broad range of analytical applications.^13,24−26^ However, as discussed above, the hydrophilic nature and porosity of cellulose substrates hampers their applicability with colloidal solutions.^26^ Hydrophobic cellulose substrates have been reported to overcome these drawbacks, enabling concentration of NPs in a small area. This strategy results in enhanced sensitivity and reproducibility, being exploited mainly in SERS analysis.^18,26,27^ In addition, some hydrophobic and super-omniphobic materials have demonstrated their potential usefulness for the development of droplet-based optical assays with enhanced sensitivity due to the increased droplet height (i.e., path length).^28−30^ In light of the above, cellulose substrates with significant water resistance could be particularly attractive as drop holders for both in-drop enrichment and plasmonic sensing purposes. To be applicable, solid substrates must be compatible with the microdrop of colloidal solution and display enough solid–liquid adhesion to prevent hanging drops from rolling off from the substrate. The latter requirement limits the use of super-omniphobic paper substrates with this aim, which nevertheless show much promise for colorimetric sensing.^30^

To the best of our knowledge, this is the first work in which waterproof cellulose-based substrates are assessed as feasible surfaces to perform in-drop enrichment and colorimetric plasmonic sensing while integrating the different unitary steps. Particularly, *in situ* generation of volatiles and extraction and preconcentration of evolved volatiles onto a microvolume of metallic NPs can be simultaneously performed, whereas the acquisition of the analytical signal can be rapidly carried out in the substrate itself without the need to collect and transfer the enriched drop. As a proof of concept, the proposed approach has been applied to the determination of acid-labile sulfide in waters.

## Experimental Section

### Reagents and Materials

All reagents were of analytical reagent grade. High-purity deionized water produced from a Millipore Sigma Simplicity ultrapure water system (Millipore Iberian S.A., Madrid, Spain) was used throughout. Tetrachloroauric acid trihydrate, trisodium citrate, formaldehyde, ascorbic acid, and hexadecyltrimethylammonium bromide (CTAB) from Sigma-Aldrich (St. Louis, MO, USA); ammonia solution (25–28%) and sodium borohydride from Merck (Darmstadt, Germany); sodium hydroxide from AnalaR Normapur, VWR (Leuven, Belgium); and silver nitrate from Scharlau (Barcelona, Spain) were used for the synthesis of NPs. The following chemicals were employed for the screening of potential analytes: potassium bromide (Fluka Chemie, Buchs, Switzerland), potassium bromate (Probus, Badalona, Spain), potassium iodide (Sigma-Aldrich), sodium sulfide trihydrate (VWR), sodium sulfite (Sigma-Aldrich), sodium nitrite (Panreac, Barcelona, Spain), ammonium nitrate (Probus), monomethylamine (MMA), dimethylamine (DMA), and trimethylamine (TMA), as hydrochlorides (Sigma-Aldrich), arsenic trioxide (Merck), antimony trichloride (Carlo Erba, Milan, Italy), mercury chloride (Prolabo, Paris, France), hydrochloric acid 37% (m/m) (Panreac), sulfuric acid 95% (m/m) (AnalaR Normapur VWR), and hydrogen peroxide 30% (m/m) (Merck). Besides, sodium thiosulfate pentahydrate (Panreac), sodium carbonate anhydrous (Panreac), and starch (Probus) were also used for the standardization of sulfide solutions by the iodometric method.^31^

Three inexpensive waterproof cellulose-based substrates were considered in this work. Whatman 1PS Phase Separator, consisting of a filter paper impregnated with a stabilized silicone typically used for phase separation, was purchased from Whatman (Maidstone, Kent, UK); polyethylene-coated filter paper, used for protecting laboratory benches from spills and leaks, was obtained from Filter Lab (Barcelona, Spain); tracing paper, suitable for graphic design purposes, was purchased at a local stationery store. Whatman No. 1 filter paper (Whatman) was also employed for comparison purposes.

### Apparatus

A Samsung Galaxy A70 smartphone (Samsung, Seoul, South Korea) and a portable PULUZ photo studio lightbox (Shenzhen PULUZ Technology Limited, Shenzhen, China) equipped with 20 LEDs were used for digitization of microdrops of colloidal solutions. RGB Color Detector App (The Programmer, Google Play Store) and ImageJ, a free image processing and analysis software,^32^ were used for data acquisition. The statistical package Statgraphics Centurion XVI.I (StatPoint Technologies, Warrengton, VA, USA) was employed for optimization purposes. With the aim of making comparative studies, a Xerox ColorQube 8580 printer (Rochester, New York, USA) and a Phoenix instrument RSM-02HP+ magnetic stirring hotplate (Garbsen, Germany) were also employed to define detection areas on hydrophilic cellulose substrates by wax-printing.^33^

A Thermo Scientific Nanodrop ND-1000 microvolume UV–vis spectrophotometer (Wilmington, DE, USA) and a JEOL JSM-1010 (Tokyo, Japan) transmission electronic microscope with an acceleration voltage of 100 kV were used to characterize NPs in the absence and presence of volatile derivatives of target analytes. Drops of colloidal solutions were deposited onto carbon-coated copper grids and air-dried before TEM analysis.

### Preparation of Colloidal Solutions

Spherical AuNPs, rod-shaped AuNPs, and Au@Ag core–shell NPs have been prepared in accordance with synthetic procedures reported in the literature.^34−38^ Detailed procedures are provided in the Supporting Material.

### Experimental Procedure for Acid-Labile Sulfide Determination

10 mL of aqueous solution (i.e., blank, standard or aqueous sample) was placed into a glass vial containing a stir bar. A 2.0-cm-diameter circular piece of a waterproof cellulose-based substrate, typically Whatman 1PS, was placed inside a screw cap over a Teflon-faced septum, and 10 μL of Au@AgNPs was deposited onto the water-resistant cellulose substrate before sealing the vial. Then, H_2_S was generated *in situ* by externally injecting 1.0 mL of HCl 1.0 M, and the hanging microdrop was exposed to the headspace above the aqueous solution stirred at 1200 rpm for a prescribed time. Finally, a smartphone was used for digitization of the microdrop and the analytical response (mean color intensity difference (Δ*I*_c_) in the B color channel, calculated by subtracting the mean color intensity of a blank to the mean color intensity of a standard or sample) was obtained.

## Results and Discussion

### Assessment of NPs Stability on Waterproof Cellulose-Based Substrates

The stability of different colloidal solutions on waterproof cellulose-based substrates was first assessed. The potential applicability of NPs as receptors for volatile sensing depends largely on their stability on the cellulose-based substrates used as drop holders. With the time-dependent nature of microextraction processes kept in mind, results derived from these studies are of paramount relevance. Thus, 5 μL of three different types of plasmonic NPs, namely, spherical AuNPs, gold nanorods (AuNRs), and Au@AgNPs, were deposited onto three inexpensive substrates, namely, Whatman 1PS, tracing paper, and polyethylene-coated filter paper. Images of the microdrops of colloidal solutions were obtained at regular intervals in the range of 0–30 min and analyzed by ImageJ. Figures S1–3 show the results derived from these studies. The stability of microdrops of colloidal solutions deposited onto cellulose-based substrates was highly dependent on the types of nanomaterials and substrates considered. In general terms, both AuNRs and Au@AgNPs showed a remarkable stability in the studied time interval, whereas the color changes produced within relatively short deposition times reflected the lack of stability of citrate-capped AuNPs, presumably due to ligand displacement and aggregation of AuNPs. Regarding the evaluated substrates, both Whatman 1PS and polyethylene-coated filter paper proved suitable for in-drop plasmonic sensing, since the mean color intensity of NPs in RGB channels remained steady. However, the tracing paper was found to be unsuitable with either colloidal solution attempted. The discoloration of microdrops deposited onto tracing paper can be ascribed to the increased interactions of NPs with this cellulose substrate that would lead to adsorption and presumably aggregation of NPs on the substrate.

The use of drop volumes larger than those typically used in SDME (i.e., 1–5 μL) is of particular interest in the development of in-drop enrichment/sensing assays. On one hand, larger drop volumes provide larger surface areas that can lead to extraction of higher amounts of volatiles, while at the same time, longer optical path lengths are obtained,^28^ which leads to increased sensitivity. The potential spreading of colloidal solutions hanging from suitable waterproof substrates, namely, Whatman 1PS and polyethylene-coated filter paper, was then assessed. Particularly, drop volumes of AuNPs, AuNRs, and Au@AgNPs of up to 30 μL were hanged from the waterproof substrates in agreement with the procedure described in the Experimental Section, and the resulting drop diameter (*D*) was compared with the diameter of an analogous microdrop deposited at the same time onto the corresponding substrates (*D*_0_). In general, Whatman 1PS enabled the exposure of larger drop volumes than polyethylene-coated filter paper and led to significantly lower *D*/*D*_0_ values when drop volumes beyond 10 μL were attempted (Figure S4). Remarkably, drops of colloidal solutions kept a quasi-spherical shape and displayed remarkable stability when Whatman 1PS substrates were used. As can be deduced from Figure S4, NP drop volumes of even 30 μL were found to be suitable for microextraction processes with the exception of AuNRs, which led to a significant rate of drop detachment when NP volumes beyond 20 μL were used. These effects can be attributed to the lower surface tension of CTAB-containing AuNRs drops. Remarkably, the increase in drop diameter of colloidal solutions was found to be lower than 20% in any case, regardless of the colloidal solutions considered and the substrates used. Furthermore, a good repeatability (RSD values below 2.0%) was observed in all cases when measuring the diameter of colloidal drops after the microextraction process.

The effect of drop volume (and hence, drop height) over the analytical response was also evaluated. This study was conducted by drop-casting colloidal solution volumes in the range of 2–30 μL onto Whatman 1PS. This substrate was selected on the basis of its compatibility with the colloidal solutions and its higher hydrophobicity (water contact angle of ca. 138°),^39^ which ensured longer drop heights and, as a consequence, reduced deviations from the ideal spherical configuration. As shown in Figure S5, the analytical signal, i.e., mean color intensity in the corresponding RGB channel, significantly increased upon increasing drop volume, and a quasi-linear response was observed between the analytical signal and the theoretical drop height (as an estimation of the optical path length) assuming a perfectly spherical configuration, i.e., *H* = 2 × (3*V*/4π)^1/3^, where *H* is the theoretical drop height and *V* corresponds to the drop volume.^28^ It is worth mentioning that larger volumes (e.g., 50 μL) of colloidal solutions showed significant stability when deposited onto Whatman 1PS or polyethylene-coated filter paper, thus being potentially applicable as cost-effective substrates for smartphone-based detection purposes. According to these results, Au@AgNPs and AuNRs were selected as the potential nanomaterials for sensing, whereas the hydrophobic Whatman 1PS was selected as the substrate of choice.

### Sensing of Volatiles

**Figure 1:**
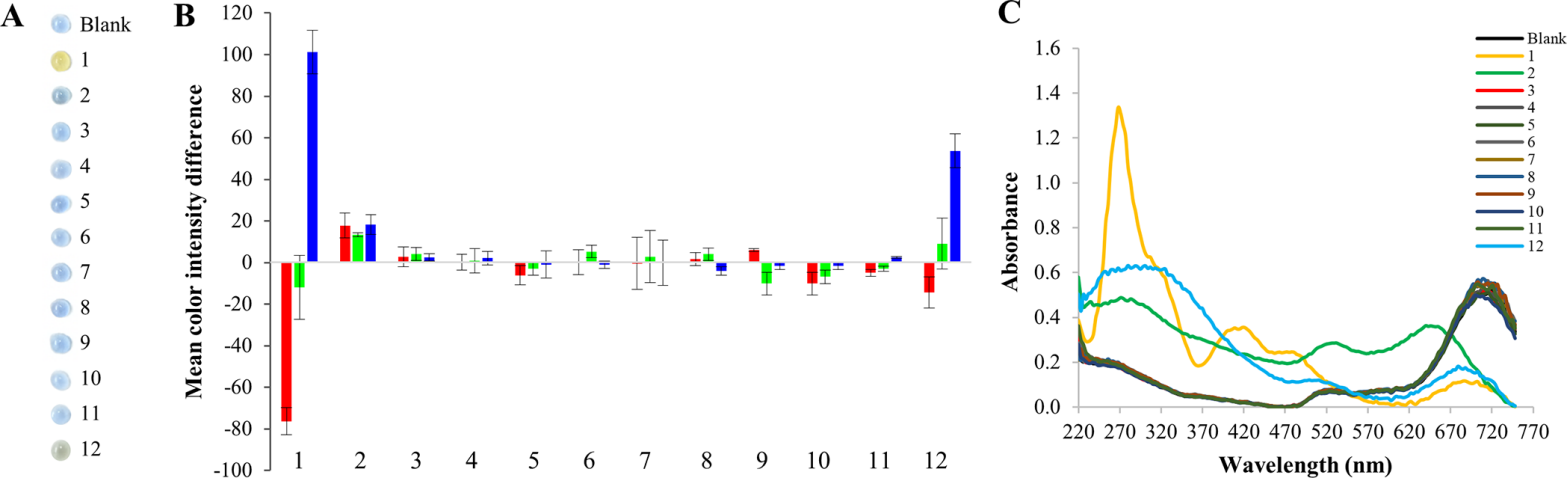
Digital images of AuNRs exposed to *in situ* generated volatiles (A). Effect of the volatiles on the analytical response (mean color intensity difference) of AuNRs in each RGB channel (B) and corresponding UV–vis spectra (C). Analytes: 1 bromide, 2 iodide, 3 sulfide, 4 sulfite, 5 nitrite, 6 ammonium, 7 monomethylammonium, 8 dimethylammonium, 9 trimethylammonium, 10 arsenic(III), 11 antimony(III), 12 mercury(II).

**Figure 2:**
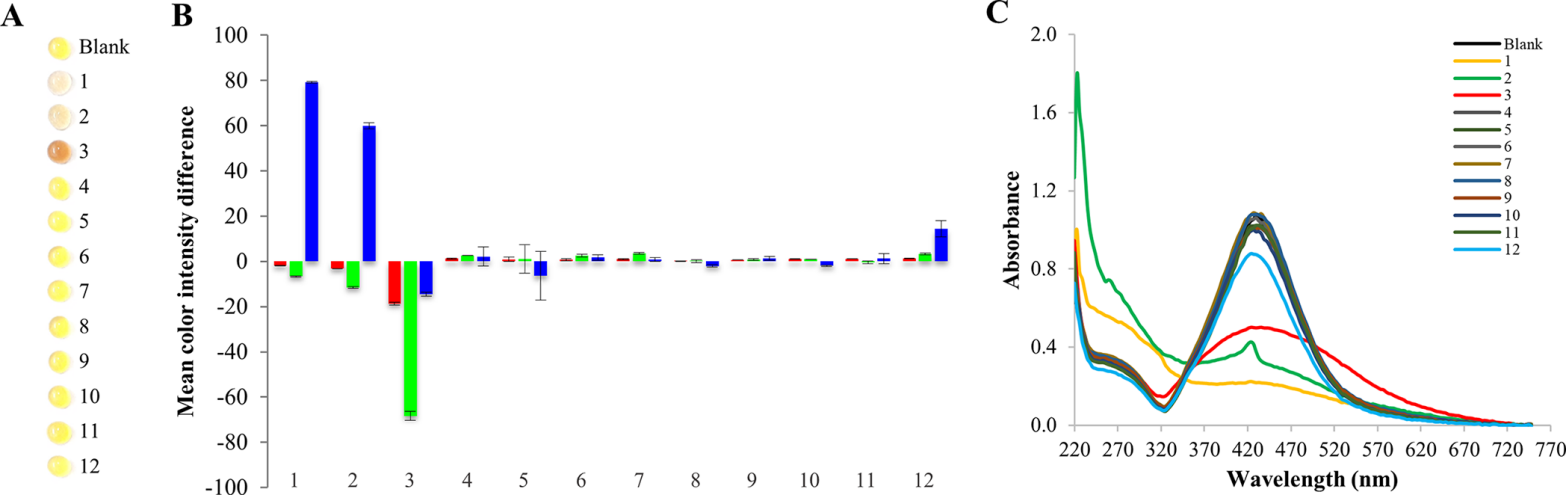
Digital images of Au@AgNPs exposed to *in situ* generated volatiles (A). Effect of the volatiles on the analytical response (mean color intensity difference) of Au@AgNPs in each RGB channel (B) and corresponding UV–vis spectra (C). Analytes: 1 bromide, 2 iodide, 3 sulfide, 4 sulfite, 5 nitrite, 6 ammonium, 7 monomethylammonium, 8 dimethylammonium, 9 trimethylammonium, 10 arsenic(III), 11 antimony(III), 12 mercury(II).

Screening of target analytes was subsequently carried out to evaluate the potential applicability of the proposed approach. Different environmentally relevant analytes, i.e., bromide, iodide, sulfide, sulfite, nitrite, ammonia, methylamine, dimethylamine, trimethylamine, arsenic(III), antimony(III), and mercury(II), were tested. The process, schematically represented in Figure S6A, involved *in situ* generation of volatile derivatives, their transfer to the headspace and trapping onto a microdrop of colloidal solution with subsequent smartphone-based colorimetric detection. Accordingly, a microvolume of Au@AgNPs or AuNRs hanging from the hydrophobic cellulose substrate (Whatman 1PS) was exposed to volatiles generated *in situ* as described elsewhere (Table S1).^6,40−45^ The digital images of AuNRs and Au@AgNPs drops before and after exposure to the volatiles, as well as the corresponding UV–vis spectra and the analytical response (mean color intensity difference in each RGB channel), are shown in Figures 1 and 2. Obtained results and possible sensing mechanisms are discussed in the sections below.

#### Sensing Studies with AuNRs

**Figure 3:**
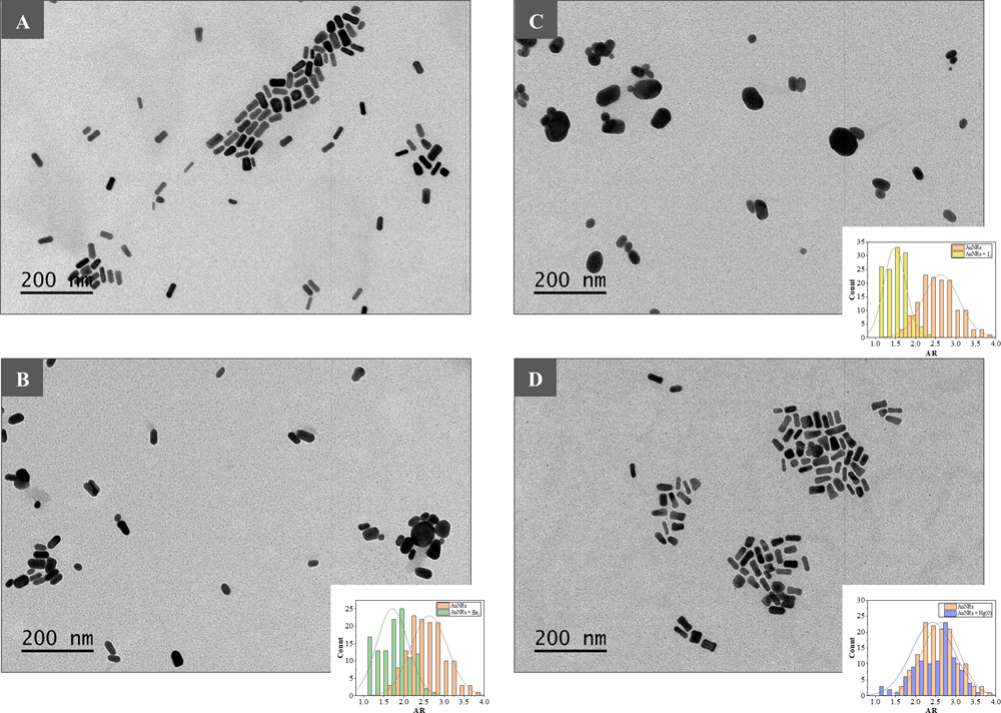
TEM images and aspect ratio distribution of AuNRs in the absence (A) and presence of Br_2_ (B), I_2_ (C), and Hg^0^ (D).

Three of the evaluated volatile analyte derivatives, i.e., Br_2_, I_2_, and Hg^0^, yielded significant effects on the color of AuNRs-containing drops that could be exploited for sensing purposes (Figure 1). In particular, an apparent color change from blue-gray to yellow was produced when AuNRs were exposed to Br_2_. This effect can be ascribed to the oxidation of Au^0^ with formation of AuBr_2_^–^ in the drop.^4,46,47^ Apart from a blue-shift and decrease in intensity of the longitudinal plasmonic band of AuNRs, the UV–vis spectra shows the appearance of the characteristic absorption peak of Br_3_^–^ (272 nm), which acts as both an oxidizing and complexing agent, together with additional bands that could be attributed to the formation of the aforementioned gold bromide complexes. Less noticeable, even though significant, color changes associated with blue-shifts of the longitudinal plasmonic band of AuNRs occurred in the presence of I_2_ and Hg^0^ that can be ascribed to oxidative etching^6,48^ and amalgamation,^5,49^ respectively. TEM images of AuNRs in the absence and presence of Br_2_, I_2_, and Hg^0^ are also provided in Figure 3. The obtained TEM images reinforced the assumption of oxidative etching and amalgamation as recognizing mechanisms for Br_2_, I_2_, and Hg^0^. As can be derived from the figure, the aspect ratio (AR) of AuNRs (2.6 ± 0.4) was significantly reduced when exposed to I_2_ (1.5 ± 0.3), whereas less noticeable effects were produced in the presence of Br_2_ (1.7 ± 0.4) and, specifically, Hg^0^ (2.4 ± 0.5). With the results shown in Figure 1B kept in mind, colorimetric sensing of Br_2_, I_2_, and Hg^0^ can be presumably carried out with AuNRs. Thus, a ratiometric sensing approach involving R and B channels could be established for Br_2_ sensing, the B channel could be employed for detection of Hg^0^, and all three channels could be employed for I_2_ sensing even though with lower sensitivity (Figure S6B).

#### Sensing Studies with Au@AgNPs

**Figure 4:**
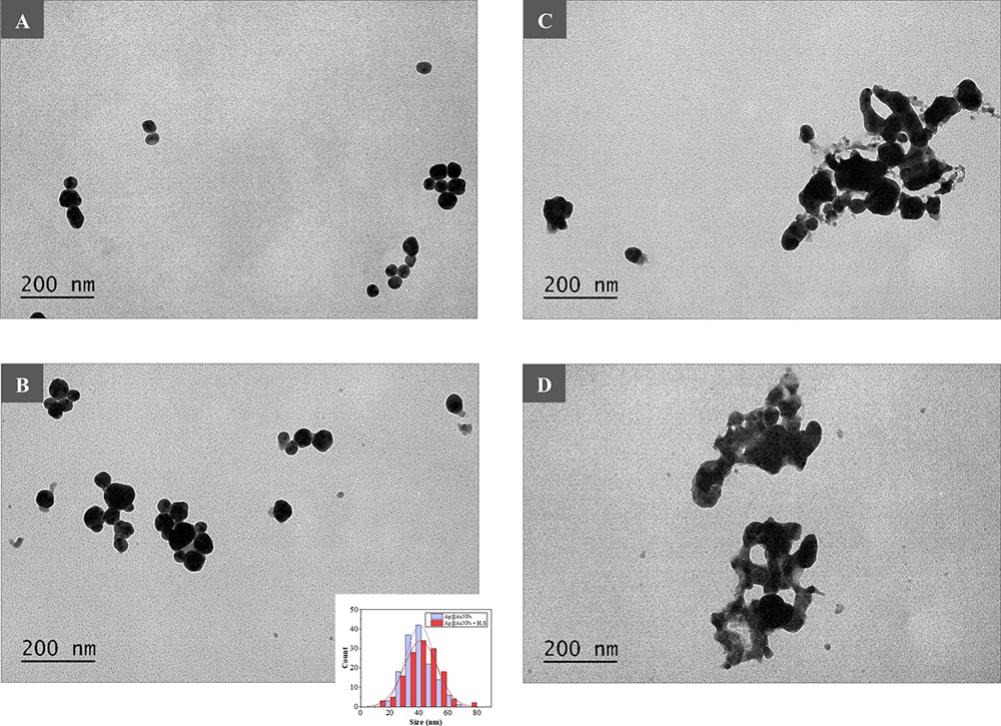
TEM images of Au@AgNPs in the absence (A) and presence of H_2_S (B), Br_2_ (C), and I_2_ (D).

Results shown in Figure 2 point out that three of the evolved volatiles, namely, H_2_S, I_2_, and Br_2_, gave rise to a significant effect on the analytical response when Au@AgNPs were used. Particularly, the exposure of Au@AgNPs to H_2_S led to a decrease, widening, and red-shift of the plasmonic band with a color change from yellow to brown under the experimental conditions used in the study (Figure 2C). Besides, Au@AgNPs droplets lost their yellow color when exposed to I_2_ and Br_2_ (Figure 2A and C). TEM images of Au@AgNPs in the absence and presence of these three volatiles are shown in Figure 4. No differences in morphology or in mean diameter (46 ± 11 nm vs 46 ± 14 nm) could be observed when Au@AgNPs were exposed to H_2_S (Figure 4B). Nevertheless, a slight variation in polydispersity (ca. 25% increase) occurred after exposure of Au@AgNPs to the volatiles. The effect of H_2_S could be attributed to the formation of Ag_2_S, as described in the literature.^23,50,51^ Unlike H_2_S, significant morphological changes of Au@AgNPs were observed when exposed to Br_2_ and I_2_ (Figure 4C and D). Particularly, both halogens led to the formation of irregular structures probably derived from the partial oxidation of the metallic NPs, adsorption of formed halides over the surface of NPs, and fusion with the formation of metallic halides.^52−54^ Regarding smartphone-based detection, the B channel enabled the detection of both bromide and iodide with higher sensitivity, whereas the highest analytical response for sulfide detection was achieved when using the G channel under the experimental conditions used in the study (Figure 2B and S6C). These results suggest that Au@AgNPs could potentially be used for H_2_S, Br_2_, and I_2_ colorimetric sensing by the proposed approach. As a proof of concept, the smartphone-based colorimetric determination of acid-labile sulfide in environmental waters was assessed in this work.

### Assessment of Experimental Parameters for In-Drop Colorimetric Sensing of H_2_S

The effect of experimental variables on both in-drop trapping of H_2_S by Au@AgNPs and smartphone-based colorimetric detection was studied. In particular, the amount of formaldehyde employed for the preparation of Au@AgNPs of increasing size in accordance with the Tollens’ reaction was first evaluated. In addition, digitization conditions, i.e., ISO and exposure value (EV), and two relevant microextraction parameters, i.e. microextraction time and drop volume, were evaluated for optimal response.

**Figure 5:**
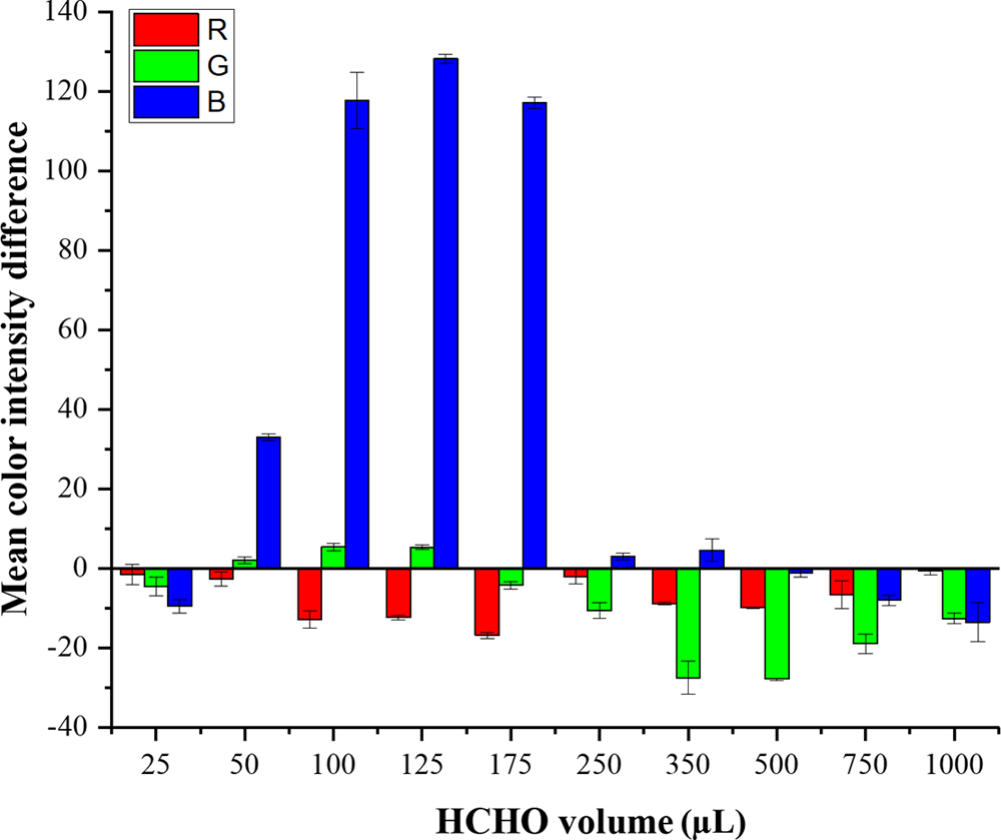
Effect of the amount of formaldehyde solution (10 mM) used for the synthesis of Au@AgNPs on the analytical response of sulfide.

The amount of formaldehyde needed to prepare Au@AgNPs in accordance with the Tollens’ reaction was first evaluated. The use of increasing amounts of formaldehyde in the presence of spherical AuNPs and Tollens’ reagent leads to the formation of Ag shells with increased thickness,^37^ which could have a paramount effect on the analytical response. Thus, Au@AgNPs with increasing dimensional sizes were prepared, and the obtained colloidal solutions were evaluated for the enrichment and sensing of H_2_S. As can be observed in Figure 5, the highest analytical response was achieved when the volume of formaldehyde solution employed for preparing Au@AgNPs was set at 125 μL, and consequently, these conditions were selected for further studies. It should be highlighted that the experimental conditions selected for the preparation of Au@AgNPs affected not only the sensitivity of the assay for sulfide determination but also the appropriate RGB color channel for data acquisition.

**Figure 6:**
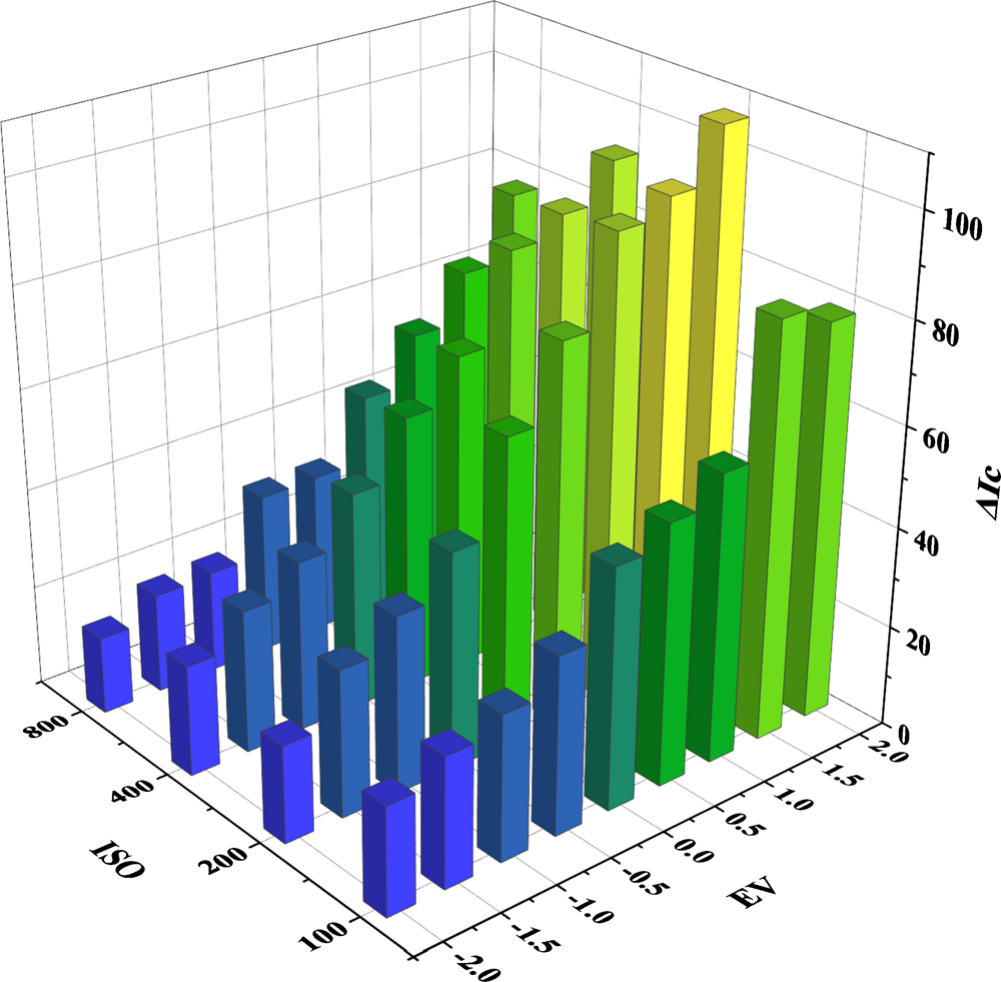
Effect of digitization conditions on the analytical response of sulfide.

Digitization conditions were subsequently evaluated. ISO sensitivity, aperture, and shutter speed values define the amount of light reaching the camera sensor, and therefore, their assessment can be of paramount importance to ensure high sensitivity for analyte detection. The ISO setting enables the adjustment of the sensor’s sensitivity to light. The higher the ISO, the higher the sensitivity to light. Besides, the EV is a combination of shutter speed and f-number, which defines the size of a lens aperture. As can be observed in Figure 6, the analytical response, Δ*I*_c_, was highly dependent on the EV and, to a lesser extent, on the ISO sensitivity. Remarkably, an appropriate selection of digitization conditions enabled a 5.5-fold enhancement of the analytical response. The EV and ISO were set at +2.0 and 200, since these conditions ensured the highest sensitivity.

**Figure 7:**
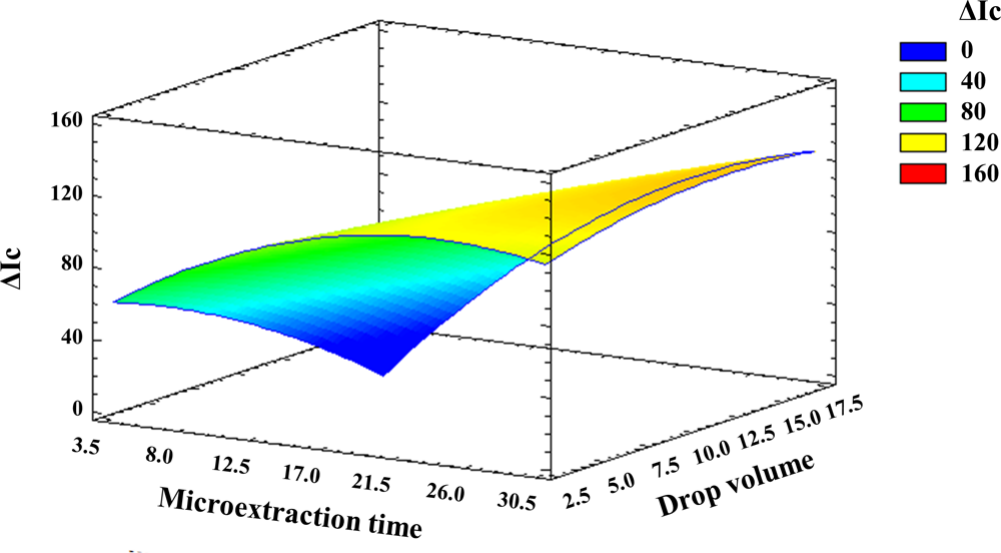
Response surface of Au@AgNPs drop volume and microextraction time.

As described in the section Assessment of NPs Stability on Waterproof Cellulose-Based Substrates, pendant droplets of 20 μL can be easily employed for in-drop enrichment/sensing with hydrophobic cellulose substrates such as Whatman 1PS, unlike those exposed to the tip of a capillary or a syringe needle, which is standard practice in SDME. Increased droplet volumes could favorably affect not only the smartphone-based detection step as a result of the increased path length, but also the extractability of the volatile, due to the increased interfacial area of colloidal microdrops. However, extended exposure times could be required when increasing the droplet radius,^55^ and partial dilution of extracted volatiles can occur, thus leading to lower enrichment factors. Thus, the combined effect of microextraction time and drop volume on the analytical response was evaluated for optimal performance. A central composite design (CCD) was selected with this aim. The levels of the experimental parameters, as well as the matrix of the CCD, are given in Table S2. As can be observed in the Pareto chart (Figure S7), both experimental parameters as well as the interaction between both variables (denoted as AB in Figure S7) and the quadratic effects (denoted as AA and BB, respectively, in Figure S7) were found to be statistically significant at the 95% confidence level. An excellent relationship between experimental data and the fitted model was obtained, with *R*^2^ = 0.99. As can be deduced from the response surface shown in Figure 7, the highest analytical response was achieved when using the longest microextraction times attempted (ca. 30 min) and drop volumes slightly above the central level (e.g., 12 μL). Thus, the possibility of using drop volumes greater than those typically used in SDME (i.e., 1–5 μL) proved to be convenient for in-drop trapping/sensing of H_2_S. The effect of Au@AgNPs drop volume on the analytical signal was highly dependent on the microextraction time used (Figure 7). Thus, the analytical response was largely dependent on the Au@AgNPs drop volume at reduced microextraction times, whereas the response was much less affected by the drop volume used when equilibrium conditions were reached. A microextraction time of 22 min and a drop volume of 10 μL were finally selected as a compromise between sensitivity and sample throughput.

### Comparison with Alternative Approaches Involving Hydrophilic Cellulose Substrates

The proposed approach was compared with alternative strategies involving hydrophilic substrates for extraction and/or smartphone-based detection (Figure S8). A hydrophilic substrate commonly used for the preparation of PADs, i.e., Whatman 1, was employed with this aim. For comparison purposes, studies were carried out with 2.0-cm-diameter circular pieces of both neat Whatman No. 1 and wax-printed Whatman No. 1 substrates with circular hydrophobic barriers defining a detection area with a diameter equivalent to the one of a drop of 10 μL of Ag@AuNPs deposited onto the hydrophobic substrate (i.e., ca. 0.27 cm). Hydrophobic barriers were fabricated as described elsewhere.^33^ First, 2.0-cm-diameter circular pieces of both hydrophilic substrates were modified with a microvolume of Au@AgNPs and employed for both extraction and smartphone-based detection of sulfide. Alternatively, microdrops of Au@AgNPs enriched with H_2_S following the experimental procedure described in the Experimental Section were deposited on Whatman No. 1 substrates (with and without hydrophobic barriers) for digitization and analytical response acquisition. As can be noted in Figure S8, the analytical response achieved with the proposed approach was found to be substantially higher than that obtained using neat Whatman 1 substrates (7.5- to 7.8-fold) and higher than that obtained with wax-printed Whatman 1 substrates (1.4- to 1.7-fold), regardless of whether they were used for both extraction and nonconventional colorimetric detection or for smartphone-based detection only. Thus, the use of Au@AgNPs with Whatman 1PS proved advantageous in terms of sensitivity and simplicity for the smartphone-based determination of sulfide.

### Analytical Performance

**Figure 8:**
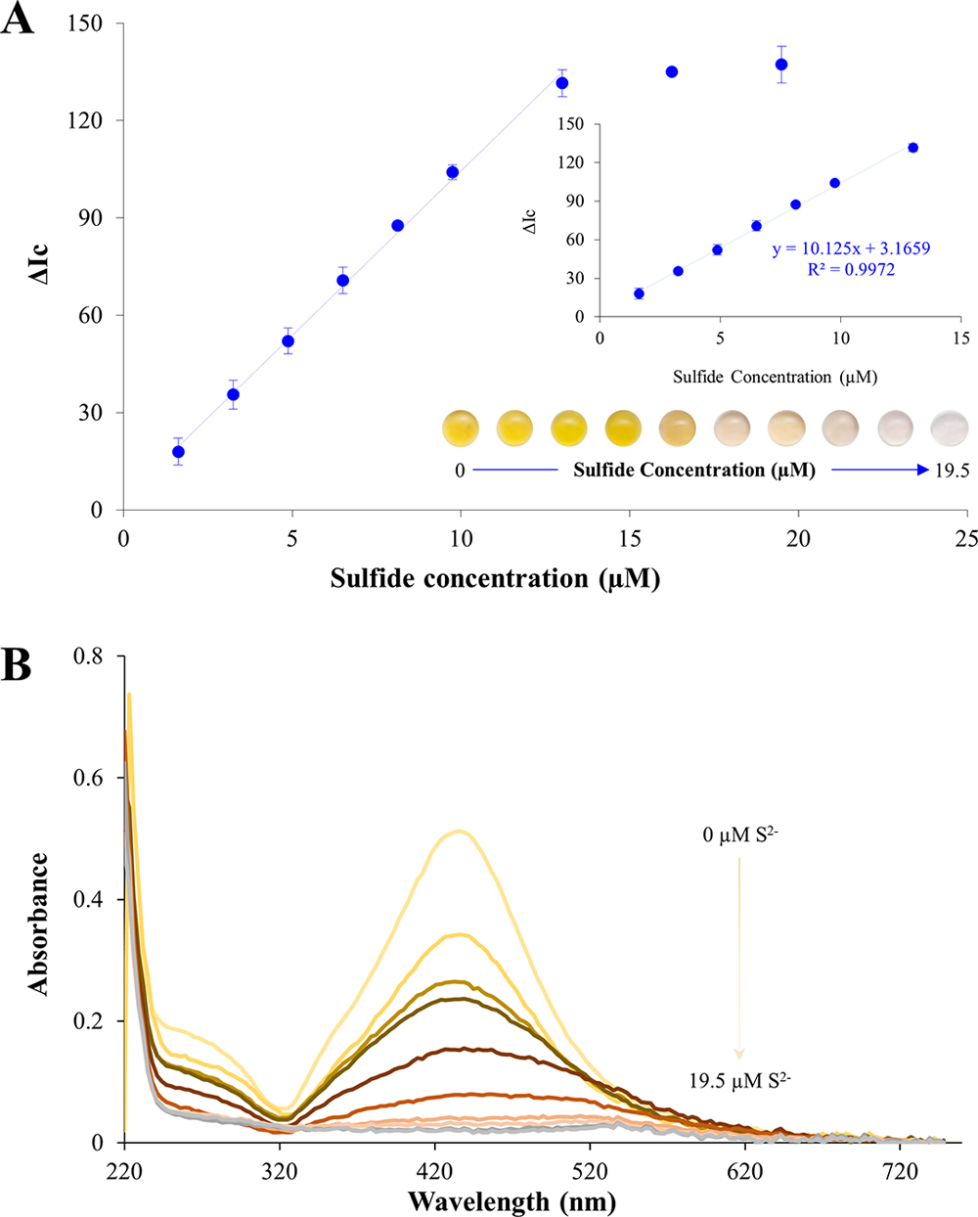
Calibration curve for sulfide determination. The inset shows the corresponding digital images of Au@AgNPs drops exposed to increasing concentrations of H_2_S (A). UV–vis spectra of Au@AgNPs drops exposed to increasing concentrations of H_2_S (B).

Under optimal conditions, the analytical performance for sulfide determination was evaluated. As can be observed in Figure 8A, the analytical response increased linearly with the concentration of the anion in the range of 1.6–13.0 μM, in agreement with the decrease of the characteristic peak of silver shell observed in the UV–vis spectra of Au@AgNPs drops exposed to increasing concentrations of H_2_S (Figure 8B).

The limits of detection (LOD) and quantification (LOQ), calculated in accordance with the 3σ criterion, were found to be 0.46 μM and 1.5 μM, respectively. The repeatability, expressed as relative standard deviation (RSD), was 4.4% (*N* = 8). A comparison of analytical characteristics of the approach with previously described methods for H_2_S colorimetric sensing involving IT equipment is provided in Table 1.

**Table 1:** Comparison of Analytical Characteristics of Colorimetric Approaches Involving IT Equipment for H_2_S Sensing

sensing material	substrate type	configuration	LOD (μM)	lowest concentration in the working range (μM)	repeatability (RSD)	refs
DMPD reagent^a^	APTES-modified chromatographic paper^b^	Test strip	4.4	12.5	2.5	(56)
DMPD reagent	Whatman filter paper grade 41	PAD	18.7	31.2	6	(57)
DMPD reagent	Whatman filter paper grade 4	μPAD	--	781	--	(58)
Cu(II)	Whatman filter paper grade 602H	PAD	0.16	3.1	6.7	(41)
Au@Ag triangular nanoplates	Agarose gel	Gel	--	31.2	--	(59)
Ag@Au nanoprisms	Eppendorf tube cap	Drop	0.065	0.1	<4.8	(23)
Au@AgNPs	Whatman 1PS	Drop	0.46	1.6	4.4	This work

a DMPD reagent, *N*,*N*′-dimethyl-*p*-phenyldiamine.

b APTES, 3-aminopropyltriethoxysilane.

A number of PADs and μPADs have been reported for H_2_S sensing, mainly based on the methylene blue formation at the detection area by reaction with the DMPD reagent,^56−58^ but also on the CuS formation by reaction with a Cu(II) salt.^41^ In addition, the potential of nanomaterials for H_2_S sensing, both incorporated into agarose gels and liquid phases,^23,59^ has been assessed in some recent contributions. The reported assay showed excellent sensitivity and precision when compared with the aforementioned alternative colorimetric methods. Notably, the LOD achieved with the proposed system was comparable to or better than that displayed by other approaches involving enrichment in both liquid drops and solid substrates, and at least 10 times better than that of DMPD-based colorimetric systems. Furthermore, the proposed approach enables exposing relatively large drop volumes to the headspace with remarkable stability, performing *in situ* H_2_S generation without loses of the volatiles, and straightforward digitization and analysis of the microdrop. In addition, modification of the assessed waterproof substrates is not required for optimal performance, unlike in other approaches involving hydrophilic substrates. In this sense, functionalization of chromatographic paper with 3-aminopropyltriethoxysilane (APTES) followed by modification with the sensing materials was required to minimize the heterogeneity of the colored product upon reaction with H_2_S,^56^ whereas hydrophobic barriers were commonly required to fabricate PADs and μPADs.^41,58^

The applicability of the proposed method for determination of acid-labile sulfide in the environmental waters method was finally assessed, with analytical results shown in Table 2. Recoveries in the range of 91–107% were obtained in all cases, thereby showing the nonsignificant effect of matrix constituents on the analytical results.

**Table 2:** Analytical Results for the Determination of Sulfide in Water Samples

sample	found sulfide (μM)	recovery^a^
Drinking water	<LOD	92 ± 5
Fountain water	<LOD	107 ± 4
Sea water (Vao)	<LOQ	95 ± 5
Sea water (Samil)	<LOQ	91.4 ± 2.2
River water	<LOQ	97 ± 3
Lake water	<LOQ	105 ± 3
Isotonic sulfurous water^b^	199 ± 4^c^	105 ± 4

a Added sulfide concentration: 9.1 μM.

b Sulfurous mineral water of volcanic origin (Averroes, Girona, Spain).

c After appropriate dilution of the sample.

## Conclusions

In this work, the feasibility of waterproof cellulose-based substrates for the in-drop plasmonic sensing of volatiles with IT equipment was assessed. The novel approach encompasses the following features: (i) hydrophobic substrates demonstrate substantial compatibility with colloidal solutions and are particularly advantageous as drop holders, enabling the exposure of relatively large drop volumes (ca. 30 μL) for volatile enrichment with a negligible risk of detachment; (ii) the use of hydrophobic substrates enables the integration of unitary steps (i.e., *in situ* volatile generation, enrichment and interaction of the volatile with the NPs, and image acquisition), thus simplifying the process; (iii) hydrophobic substrates behave as cuvetteless drop holders during image acquisition and ensure high sensitivity thanks to the increased path length; (iv) fabrication of PADs, commonly consisting of the formation of hydrophobic barriers for defining hydrophilic detection areas, is not required, and so, the sensitivity achieved with the drop-based approach is significantly larger than that obtained with PADs. Overall, the proposed approach was demonstrated to be straightforward and highly convenient for optical sensing purposes, and further developments based on the combination of a wide range of waterproof cellulose-based substrates with optically responsive materials for in-drop enrichment and sensing purposes can be anticipated.

## Supporting Information Available

Preparation of colloidal solutions. Synthetic procedures for the preparation of AuNPs, AuNRs, and Au@AgNPs. Digital images of AuNPs, AuNRs, and Au@AgNPs deposited on waterproof cellulose substrates for 0–30 min and evolution of the color intensity of colloidal solutions in RGB channels. Evaluation of microdrop spreading (*D*/*D*_0_) at increasing volumes of AuNPs, AuNRs, and Au@AgNPs hanged from waterproof substrates in agreement with the microextraction procedure. Effect of drop volume of AuNPs, AuNRs, and Au@AgNPs on mean color intensity. Schematic representation of the system used for in-drop enrichment/sensing of volatiles and effect of *in situ* generated volatiles on the appearance of AuNRs and Au@AgNPs. Pareto chart of the main effects obtained from the central composite design. Comparison of the proposed approach with alternatives involving hydrophilic cellulose substrates for sulfide determination. Experimental conditions for *in situ* generation of volatile derivatives. Experimental factors, levels evaluated, and matrix of the CCD. (PDF)
