# Supplementary material for: Waterproof Cellulose-Based Substrates for In-Drop Plasmonic Colorimetric Sensing of Volatiles: Application to Acid-Labile Sulfide Determination in Waters
Source: ACS Sens. 2022 Mar 14;7(3):839–48. doi: 10.1021/acssensors.1c02585 (PMC8961881; doi:10.1021/acssensors.1c02585)
Supplement: Supplementary file 1 — se1c02585_si_001.pdf [file se1c02585_si_001.pdf]

# Waterproof Cellulose-Based Substrates for In-Drop Plasmonic Colorimetric Sensing of Volatiles: Application to Acid-Labile Sulfide Determination in Waters

**Nerea Villarino, Francisco Pena-Pereira*, Isela Lavilla, Carlos Bendicho***

*Centro de Investigación Mariña, Universidade de Vigo, Departamento de Química Analítica e alimentaria, Grupo QA2, Edificio CC Experimentais, Campus de Vigo, As Lagoas, Marcosende 36310 Vigo, Spain.*

*E-mail addresses:* fjpena@uvigo.es; bendicho@uvigo.es

## Supplementary Material

# Table of contents


**Preparation of colloidal solutions**

**Figure S1.** Digital images of AuNPs deposited on waterproof cellulose substrates for 0-30 min. Evolution of the color intensity of AuNPs after 0-30 min in RGB channels (B-D). Experimental conditions: Initial AuNPs volume, 5 µL; digitization conditions, PRO ISO 100, exposure value, +2.0.

**Figure S2.** Digital images of AuNRs deposited on waterproof cellulose substrates for 0-30 min. Evolution of the color intensity of AuNRs after 0-30 min in RGB channels (B-D). Experimental conditions: Initial AuNRs volume, 5 µL; digitization conditions, PRO ISO 100, exposure value, +2.0.

**Figure S3.** Digital images of Au@AgNPs deposited on waterproof cellulose substrates for 0-30 min (A). Evolution of the color intensity of Au@AgNPs after 0-30 min in RGB channels (B-D). Experimental conditions: Initial Au@AgNPs volume, 5 µL; digitization conditions, PRO ISO 100, exposure value, +2.0.

**Figure S4.** Evaluation of microdrop spreading ($D/D_0$) at increasing volumes of AuNPs (A), AuNRs (B) and Au@AgNPs (C) hanged from waterproof substrates in agreement with the microextraction procedure.

**Figure S5.** Effect of drop volume of AuNPs (A), AuNRs (B) and Au@AgNPs (C) on mean color intensity.

**Figure S6.** Schematic representation of the system used for in-drop enrichment/sensing of volatiles (A). Effect of *in situ* generated volatiles on the appearance of AuNRs (B) and Au@AgNPs (C).

**Figure S7**: Pareto chart of the main effects obtained from the central composite design.

**Figure S8.** Comparison of the proposed approach with alternatives involving hydrophilic cellulose substrates for sulfide determination. Experimental details: Microextraction of $H_2S$ by a drop of Au@AgNPs using Whatman 1PS as holder and subsequent deposition of enriched Au@AgNPs on neat Whatman No. 1 for smartphone-based detection (1). Extraction of $H_2S$ by a drop of Au@AgNPs using Whatman 1PS as holder and subsequent deposition of enriched Au@AgNPs on the detection area of wax-printed Whatman No. 1 for smartphone-based detection (2). Microextraction and smartphone-based detection involving neat Whatman No. 1 containing Au@AgNPs (3). Microextraction and smartphone-based detection involving wax-printed Whatman No. 1 containing Au@AgNPs on its detection area (4). Proposed approach involving a drop of Au@AgNPs and Whatman 1PS (5).

**Table S1**. Experimental conditions for *in situ* generation of volatile derivatives.

**Table S2**. Experimental factors, levels evaluated and matrix of the CCD.

## Preparation of colloidal solutions

### *Synthesis of citrate-capped AuNPs*[1,2]

2.5 mL of a 39 mM trisodium citrate solution was rapidly added to a 25 mL of a 1 mM tetrachloroauric acid solution vigorously boiled and stirred in a round-bottom flask with a reflux condenser. The solution was heated and refluxed for an additional 15 min and subsequently cooled to room temperature while stirring.

### *Synthesis of AuNRs*[3]

AuNRs were prepared by a seed-mediated growth method. Seeds were firstly prepared at 28 °C. In brief, 25 µL of 50 mM tetrachloroauric acid was added to 4.7 mL of 100 mM CTAB and slowly stirred for 5 min. Then, 300 µL of ice-cold, freshly prepared 10 mM sodium borohydride solution was added all at once with vigorous stirring. After 20 s, the stirring rate was adjusted to 400 rpm and the resulting seed solution was used to obtain AuNRs.

In another vial, 100 µL of 50 mM tetrachloroauric acid was added to 10 mL of 100 mM CTAB and the resulting solution was slowly stirred at 27 °C. After 10 min, 75 µL of 100 mM ascorbic acid and 80 µL of 5 mM silver nitrate were added to the stirred solution. Then, 150 µL of the seed solution was added to the growth solution under vigorous agitation and finally left undisturbed for 30 min. Excess CTAB was removed by centrifugation of the colloidal solution.

### *Synthesis of Au@AgNPs*[4,5]

Firstly, spherical AuNPs were prepared by the Turkevich-Frens method, as described above. Subsequently, Au@AgNPs were obtained at room temperature by sequentially mixing 500 µL of AuNPs, 3750 µL of ultrapure water, 250 µL of Tollens' reagent (prepared by mixing 1.0 mL of a 0.5 M solution of silver nitrate with 1040 µL of aqueous ammonia solution (25-28%), 650 µL of a 3 M NaOH solution and making up to 20 mL with ultrapure water) and a variable volume (e.g. 25-1000 µL) of a 10 mM solution of formaldehyde.

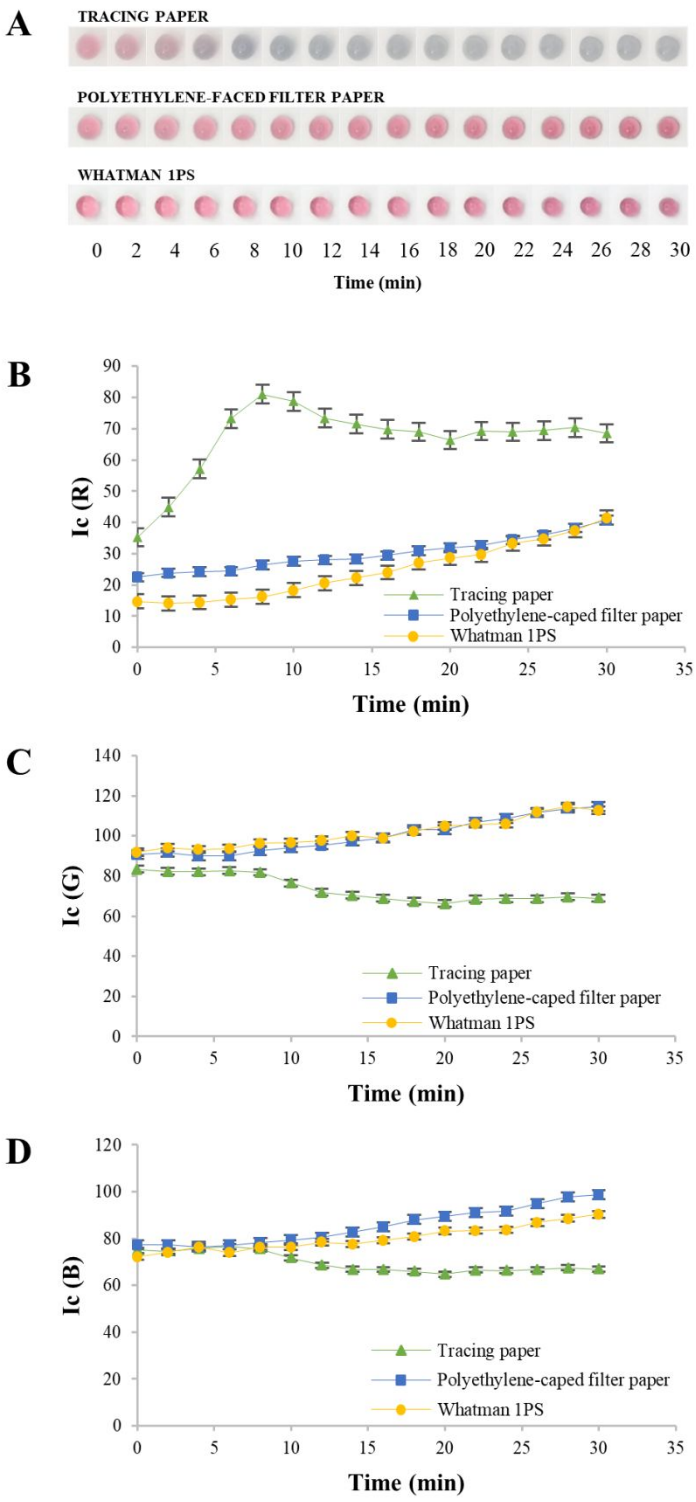


**Figure S1.** Digital images of AuNPs deposited on waterproof cellulose substrates for 0-30 min. Evolution of the color intensity of AuNPs after 0-30 min in RGB channels (B-D). Experimental conditions: Initial AuNPs volume, 5 µL; digitization conditions, PRO ISO 100, exposure value, +2.0.

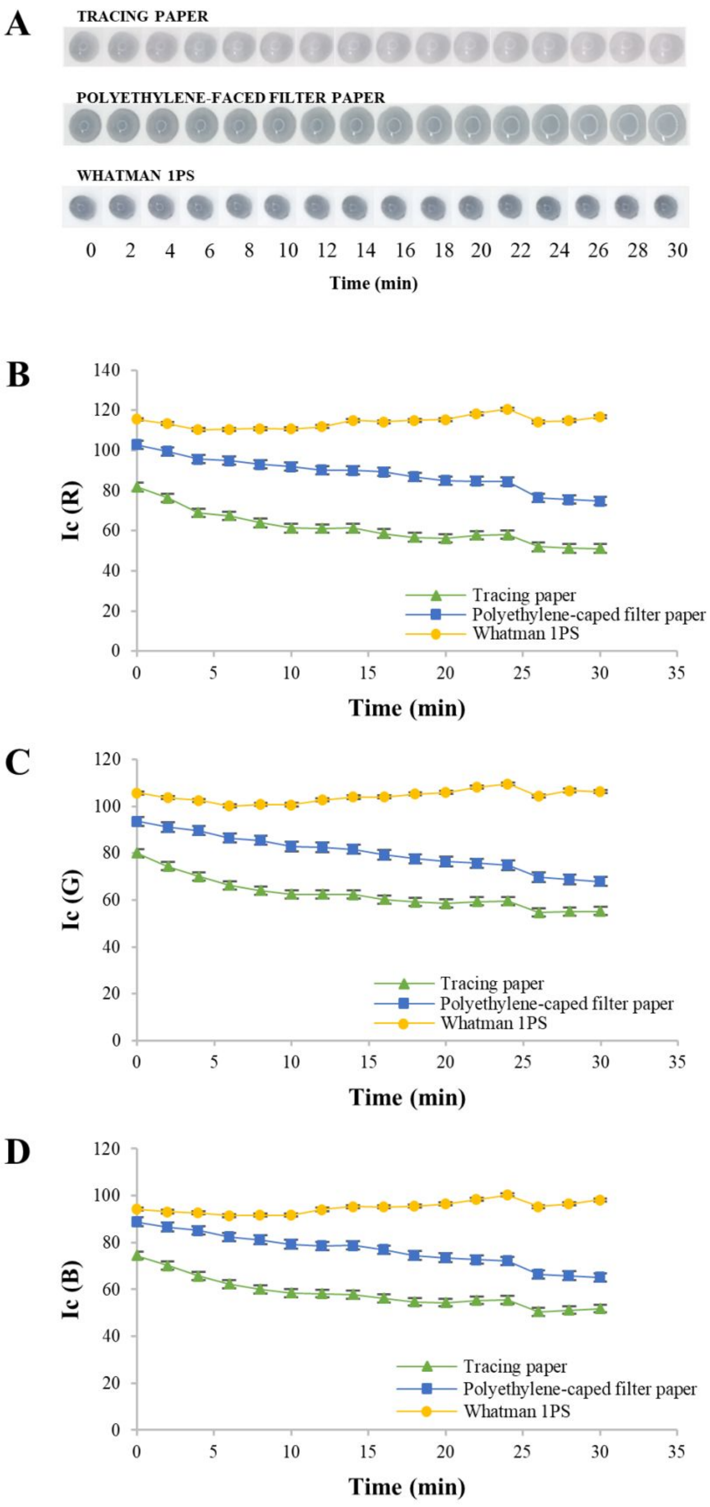


**Figure S2.** Digital images of AuNRs deposited on waterproof cellulose substrates for 0-30 min. Evolution of the color intensity of AuNRs after 0-30 min in RGB channels (B-D). Experimental conditions: Initial AuNRs volume, 5 µL; digitization conditions, PRO ISO 100, exposure value, +2.0.

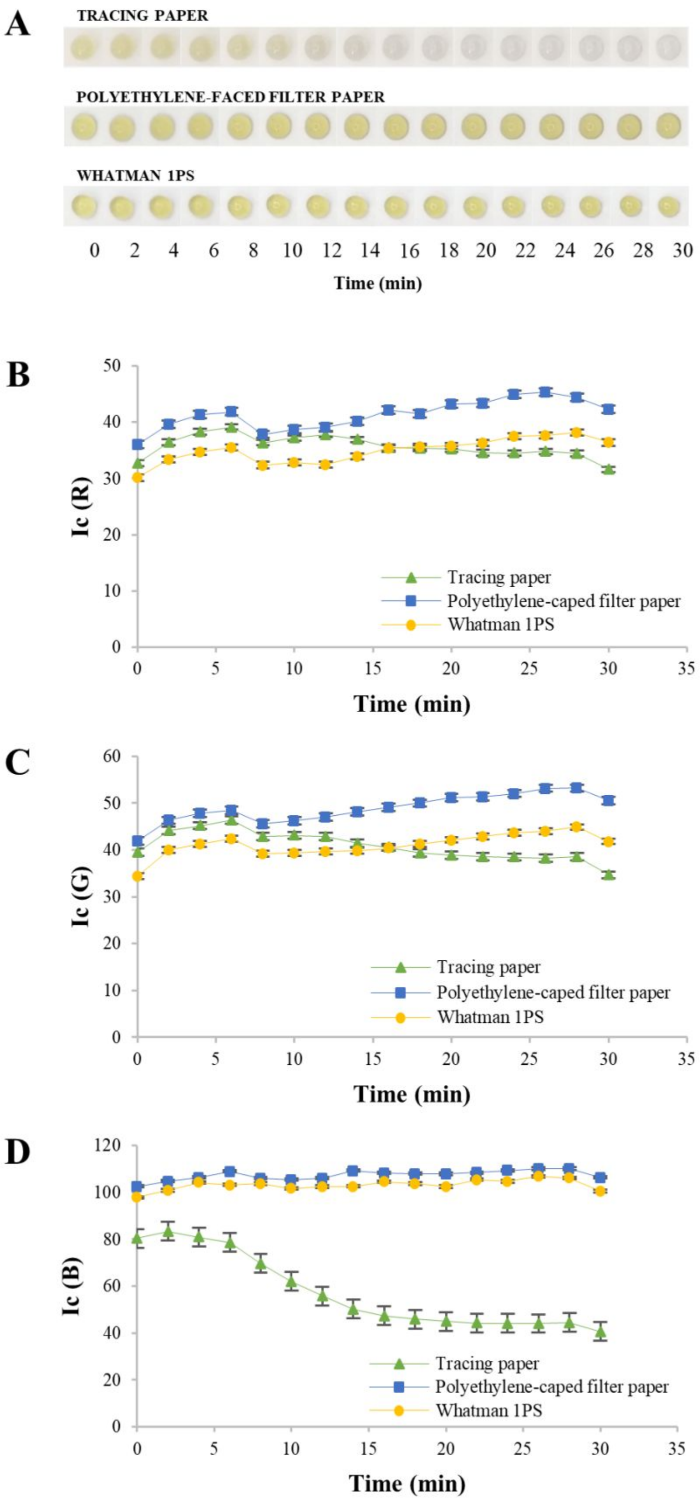


**Figure S3.** Digital images of Au@AgNPs deposited on waterproof cellulose substrates for 0-30 min (A). Evolution of the color intensity of Au@AgNPs after 0-30 min in RGB channels (B-D). Experimental conditions: Initial Au@AgNPs volume, 5 µL; digitization conditions, PRO ISO 100, exposure value, +2.0.

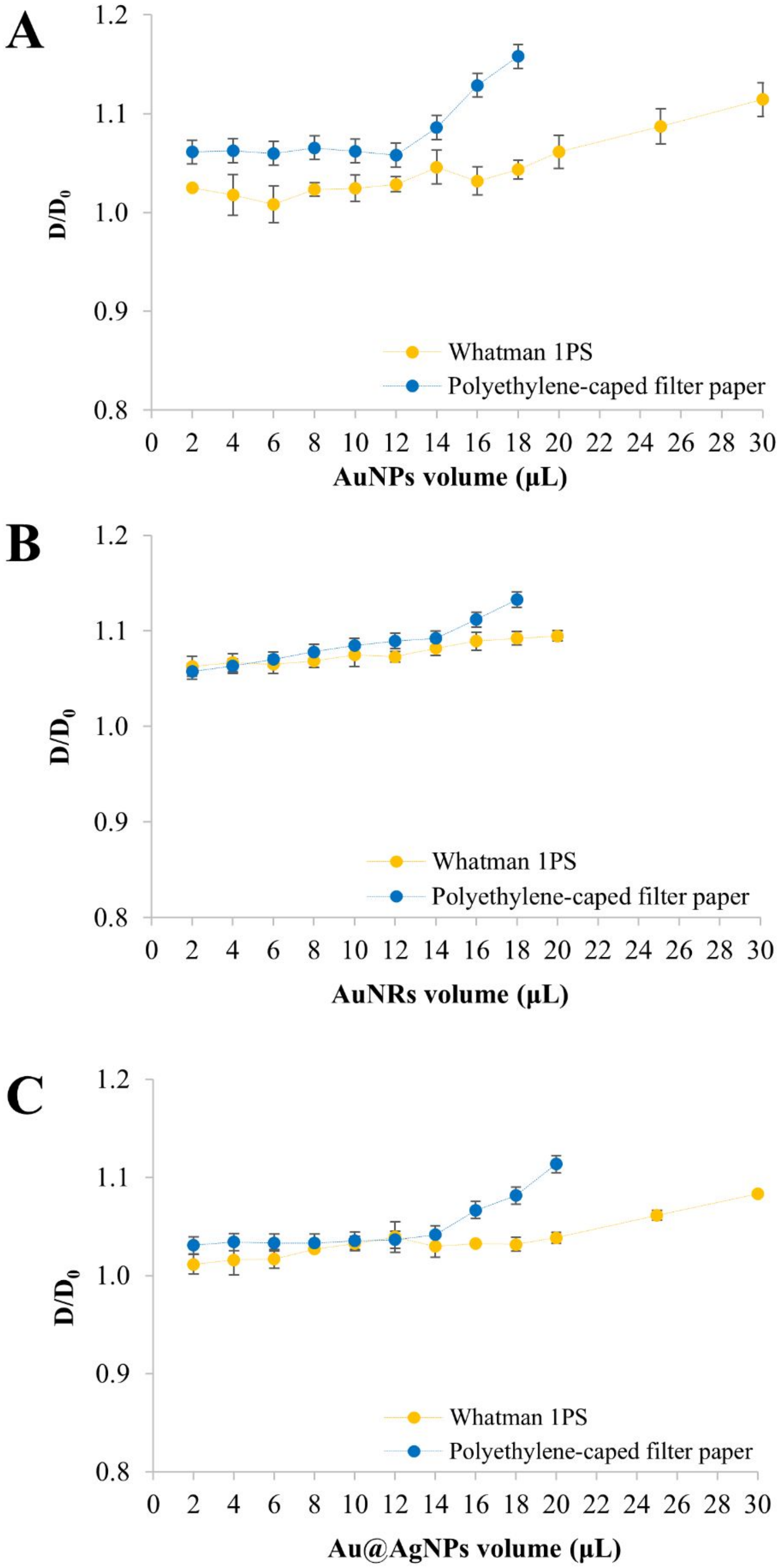


**Figure S4.** Evaluation of the microdrop spreading ($D/D_0$) at increasing volumes of AuNPs (A), AuNRs (B) and Au@AgNPs (C) hanged from waterproof substrates in agreement with the microextraction procedure.

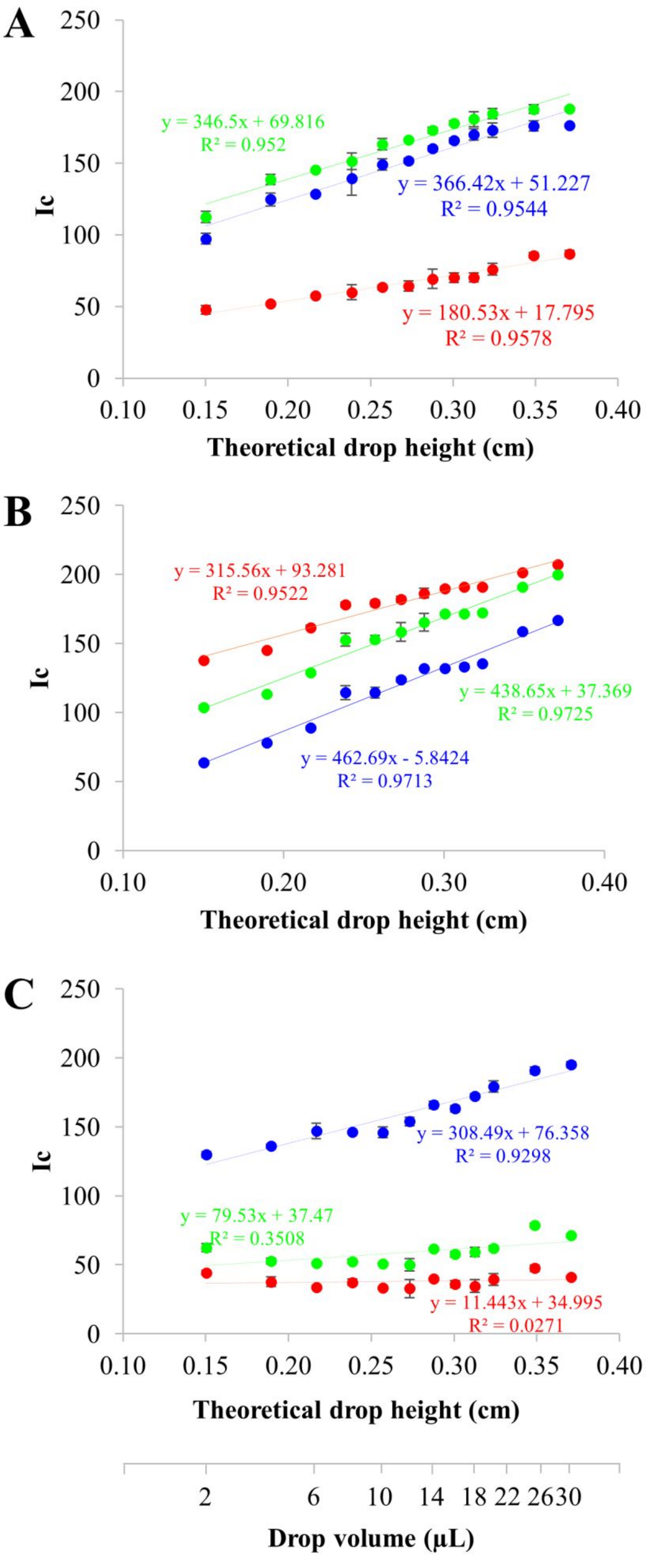


**Figure S5.** Effect of drop volume of AuNPs (A), AuNRs (B) and Au@AgNPs (C) on mean color intensity.

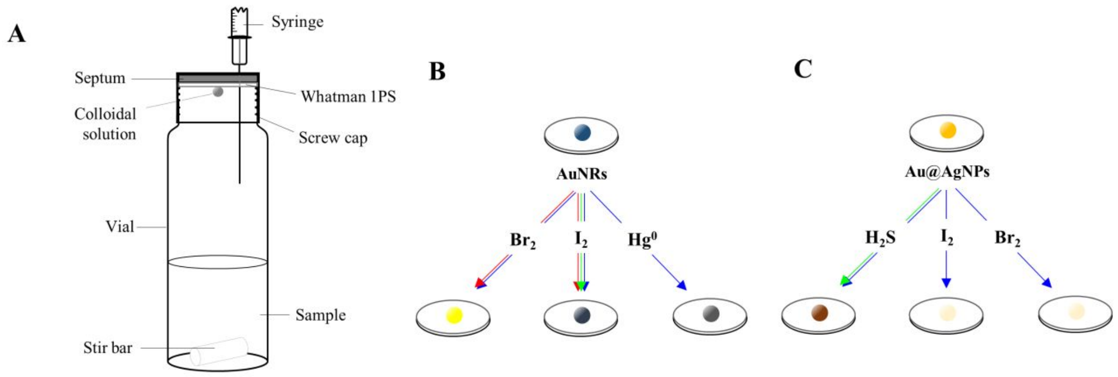


**Figure S6.** Schematic representation of the system used for in-drop enrichment/sensing of volatiles (A). Effect of *in situ* generated volatiles on the appearance of AuNRs (B) and Au@AgNPs (C).

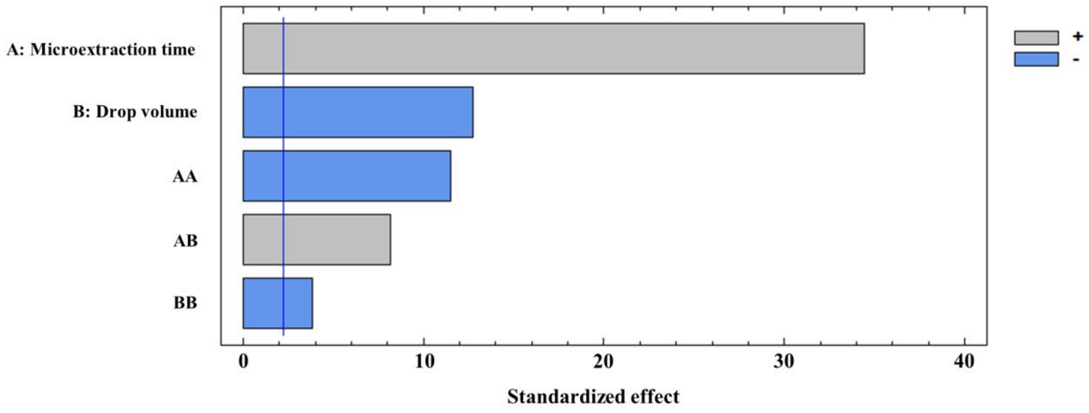


**Figure S7**: Pareto chart of the main effects obtained from the central composite design.

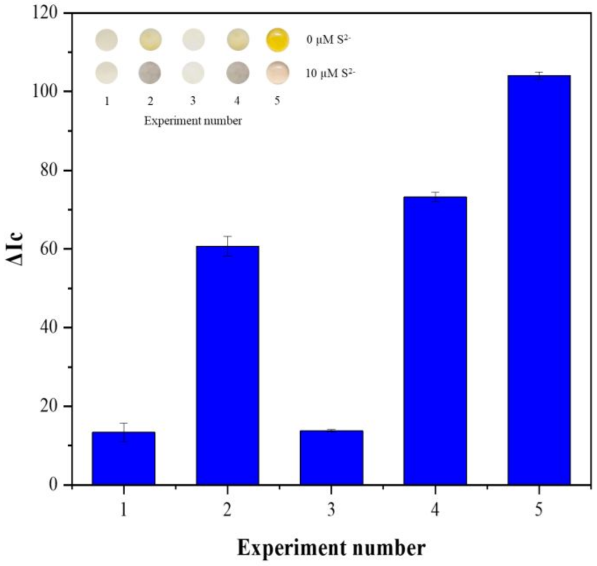


**Figure S8.** Comparison of the proposed approach with alternatives involving hydrophilic cellulose substrates for sulfide determination. Experimental details: Microextraction of $H_2S$ by a drop of Au@AgNPs using Whatman 1PS as holder and subsequent deposition of enriched Au@AgNPs on neat Whatman No. 1 for smartphone-based detection (1). Extraction of $H_2S$ by a drop of Au@AgNPs using Whatman 1PS as holder and subsequent deposition of enriched Au@AgNPs on the detection area of wax-printed Whatman No. 1 for smartphone-based detection (2). Microextraction and smartphone-based detection involving neat Whatman No. 1 containing Au@AgNPs (3). Microextraction and smartphone-based detection involving wax-printed Whatman No. 1 containing Au@AgNPs on its detection area (4). Proposed approach involving a drop of Au@AgNPs and Whatman 1PS as holder (5).

**Table S1**

Experimental conditions for *in situ* generation of volatile derivatives

| Analyte | Analyte derivative | Volatile generation conditions | Ref. |
|---|---|---|---|
| Bromide | Bromine | 1 mL $KBrO_3$ 5 mM; 10 mL $H_2SO_4$ 0.75 M | 6 |
| Iodide | Iodine | 1 mL $H_2O_2$ 1 M; 10 mL $H_2SO_4$ 2 M | 7 |
| Sulfide | Hydrogen sulfide | 1 mL HCl 1.0 M; 10 mL $H_2O$ | 8 |
| Sulfite | Sulfur dioxide | 1 mL HCl 2.5 M; 10 mL $H_2O$ | 9 |
| Nitrite | Nitrogen oxides | 1 mL HCl 1.0 M; 10 mL $H_2O$ | 8 |
| Ammonium | Ammonia | 1 mL NaOH 1 M; 10 mL $H_2O$ | 10 |
| Monomethylammonium | Monomethylamine | 1 mL NaOH 1 M; 10 mL $H_2O$ | 10 |
| Dimethylammonium | Dimethylamine | 1 mL NaOH 1 M; 10 mL $H_2O$ | 10 |
| Trimethylammonium | Trimethylamine | 1 mL NaOH 1 M; 10 mL $H_2O$ | 10 |
| Arsenic (III) | Arsine | 1 mL $NaBH_4$ 0.3% (w/v); HCl 2% (v/v) | 11 |
| Antimony (III) | Stibine | 0.1 mL $NaBH_4$ 1% (w/v); HCl 2 M | 12 |
| Mercury (II) | Mercury (0) | 1 mL $NaBH_4$ 1% (w/v); 0.01 M HCl | 11 |

**Table S2**. Experimental factors, levels evaluated and matrix of the CCD

| **Experimental factors** | **Levels** | | | | |
|---|---|---|---|---|---|
| | **-α** | **-1** | **0** | **+1** | **+α** |
| A: Microextraction time (min) | 4.3 | 8.0 | 17.0 | 26.0 | 29.7 |
| B: Ag@AuNPs drop volume (µL) | 2.9 | 5.0 | 10.0 | 15.0 | 17.1 |

| **Run** | **A** | **B** | **ΔIc**[a] |
|---|---|---|---|
| 1 | 0 | 0 | 103.9±3.0 |
| 2 | 0 | +α | 75.4±3.5 |
| 3 | 0 | 0 | 104.4±4.1 |
| 4 | -1 | +1 | 29.1±4.0 |
| 5 | -1 | -1 | 79.1±1.8 |
| 6 | 0 | 0 | 98.4±0.7 |
| 7 | +1 | +1 | 119.3±4.5 |
| 8 | 0 | -α | 109.7±5.0 |
| 9 | +α | 0 | 127.6±6.7 |
| 10 | -α | 0 | 26.8±2.8 |
| 11 | 0 | 0 | 106.0±6.1 |
| 12 | +1 | -1 | 123.0±3.7 |
| 13 | 0 | 0 | 98.9±5.4 |
| 14 | 0 | 0 | 100.4±5.3 |
| 15 | 0 | 0 | 101.4±6.1 |
| 16 | 0 | 0 | 100.2±5.8 |

[a]Average ± standard deviation, n=3

## References


(1) Huang, C.-C.; Chang, H.-T. Selective Gold-Nanoparticle-Based “Turn-on” Fluorescent Sensors for Detection of Mercury(II) in Aqueous Solution. *Anal. Chem.* **2006**, *78*, 8332–8338. https://doi.org/10.1021/ac061487i.

(2) Frens, G. Controlled Nucleation for the Regulation of the Particle Size in Monodisperse Gold Suspensions. *Nat. Phys. Sci.* **1973**, *241*, 20–22. https://doi.org/10.1038/physci241020a0.

(3) Scarabelli, L.; Sánchez-Iglesias, A.; Pérez-Juste, J.; Liz-Marzán, L. M. A “ Tips and Tricks ” Practical Guide to the Synthesis of Gold Nanorods. *J. Phys. Chem. Lett.* **2015**, *6*, 4270–4279. https://doi.org/10.1021/acs.jpclett.5b02123.

(4) Zeng, J. Bin; Fan, S. G.; Zhao, C. Y.; Wang, Q. R.; Zhou, T. Y.; Chen, X.; Yan, Z. F.; Li, Y. P.; Xing, W.; Wang, X. D. A Colorimetric Agarose Gel for Formaldehyde Measurement Based on Nanotechnology Involving Tollens Reaction. *Chem. Commun.* **2014**, *50* (60), 8121–8123. https://doi.org/10.1039/c4cc00914b.

(5) Zeng, J. Bin; Cao, Y. Y.; Chen, J. J.; Wang, X. D.; Yu, J. F.; Yu, B. Bin; Yan, Z. F.; Chen, X. Au@Ag Core/Shell Nanoparticles as Colorimetric Probes for Cyanide Sensing. *Nanoscale* **2014**, *6*, 9939–9943. https://doi.org/10.1039/c4nr02560a.

(6) García-Figueroa, A.; Pena-Pereira, F.; Lavilla, I.; Bendicho, C. Headspace Single-Drop Microextraction Coupled with Microvolume Fluorospectrometry for Highly Sensitive Determination of Bromide. *Talanta* **2017**, *170*, 9–14. https://doi.org/10.1016/j.talanta.2017.03.090.

(7) Pena-Pereira, F.; Lavilla, I.; Bendicho, C. Unmodified Gold Nanoparticles for In-Drop Plasmonic-Based Sensing of Iodide. *Sensors Actuators B. Chem.* **2017**, *242*, 940–948. https://doi.org/10.1016/j.snb.2016.09.161.

(8) Pena-Pereira, F.; Matesanz, Ó.; Lavilla, I.; Bendicho, C. A Paper-Based Gas Sensor for Simultaneous Noninstrumental Colorimetric Detection of Nitrite and Sulfide in Waters. *J. Sep. Sci.* **2020**, *43*, 1908–1914. https://doi.org/10.1002/jssc.201901339.

(9) Gómez-Otero, E.; Costas, M.; Lavilla, I.; Bendicho, C. Ultrasensitive, Simple and Solvent-Free Micro-Assay for Determining Sulphite Preservatives (E220-228) in Foods by HS-SDME and UV-Vis Micro-Spectrophotometry Microextraction Techniques. *Anal. Bioanal. Chem.* **2014**, *406*, 2133–2140. https://doi.org/10.1007/s00216-013-7293-3.

(10) Pena-Pereira, F.; Lavilla, I.; Bendicho, C. Colorimetric Assay for Determination of Trimethylamine-Nitrogen (TMA-N) in Fish by Combining Headspace-Single-Drop Microextraction and Microvolume UV-Vis Spectrophotometry. *Food Chem.* **2010**, *119*, 402–407. https://doi.org/10.1016/j.foodchem.2009.07.038.

(11) Costas-Mora, I.; Romero, V.; Pena-Pereira, F.; Lavilla, I.; Bendicho, C. Quantum Dot-Based Headspace Single-Drop Microextraction Technique for Optical Sensing of Volatile Species. *Anal. Chem.* **2011**, *83*, 2388–2393. https://doi.org/10.1021/ac103223e.

(12) Pena-Pereira, F.; Lavilla, I.; Bendicho, C. Headspace Single-Drop Microextraction with in Situ Stibine Generation for the Determination of Antimony (III) and Total Antimony by Electrothermal-Atomic Absorption Spectrometry. *Microchim. Acta* **2009**, *164* (1–2), 77–83. https://doi.org/10.1007/s00604-008-0036-z.
